# Dosimetric commissioning of a high-resolution CMOS 2D detector array for patient-specific QA of single-isocenter multi-target VMAT stereotactic radiosurgery

**DOI:** 10.1093/jrr/rrae080

**Published:** 2024-10-15

**Authors:** Ching-Ling Teng, Shih-Chi Lin, Dale Michael Lovelock, Seng Boh Lim

**Affiliations:** Radiation Oncology, Mount Sinai West, 1000 10th Avenue, New York, NY 10019, USA; Biomedical Engineering, One University Avenue, University of Massachusetts Lowell, Lowell, MA, 01854, USA; Medical Physics, Memorial Sloan-Kettering Cancer Center, 1275 York Avenue, New York, NY 10065, USA; Radiation Oncology, The Mount Sinai Hospital, 1158 5th Avenue, New York, NY 10029, USA; Medical Physics, Memorial Sloan-Kettering Cancer Center, 1275 York Avenue, New York, NY 10065, USA

**Keywords:** patient-specific QA, myQA SRS, CMOS 2D diode array, single isocenter multiple target SIMT, stereotactic radiosurgery SRS, radiochromic film dosimetry

## Abstract

Stereotactic radiosurgery (SRS) using the single-isocenter-multiple-target (SIMT) technique by volumetric modulated arc therapy is increasingly popular for treating multiple brain metastases. However, the complex nature of SIMT SRS necessitates rigorous patient-specific quality assurance (PSQA). This study presents a multi-institutional dosimetric commissioning of a high-resolution complementary metal oxide semiconductor (CMOS) 2D detector array, the myQA SRS device for SIMT SRS PSQA. Basic dosimetric properties such as dose-rate, field-size, energy and angular dependencies were characterized for the CMOS detectors. Additionally, gamma index analyses were performed between the measured dose and the films for nine simulated and clinical plans. The results showed that the CMOS detector was dose-rate, field-size, energy and beam-angle dependent. Specific to SIMT SRS, angular dependence on gantry rotations was invariant to couch rotations but was sensitive to off-isocenter distances. With appropriate dose calibration and angular corrections, myQA SRS showed a high dosimetric correlation with films. The average gamma index pass rates were 99.9 ± 0.03% and 99.2 ± 1.1% at 3%/2 mm/10%thr(global) and 1 mm/1%/10%thr(local) criteria, respectively. The average dose difference between myQA SRS and films was 0.4 ± 1.3%. In conclusion, the CMOS 2D detector array has demonstrated its potential as a reliable tool for PSQA for SIMT SRS. The excellent dosimetric agreement with the films was consistent in multiple institutions, further validating the dosimetric accuracy and reproducibility. It provides a timely alternative to film dosimetry for commissioning and quality assurance.

## INTRODUCTION

Volumetric modulated arc therapy (VMAT) for stereotactic radiosurgery (SRS) has increasingly become the standard of care for patients with multiple brain metastases [1, 2]. An important technical advancement in managing multiple brain metastases has been the introduction of the single-isocenter-multiple-target (SIMT) technique [3, 4]. Using multi-leaf collimators (MLCs) in a C-arm linear accelerator (LINAC), the SIMT technique allows simultaneous treatment of multiple lesions with a single plan and delivery. When used with FFF beams, the high dose rates further reduce treatment time and improve patient experiences [5].

However, implementing SIMT is more challenging than the traditional single-isocenter-single-target (SIST) technique. The gantry-defined radiation isocenter must be in tight concordance with all rotational axes to minimize the size of the isocenter. The rotational errors could increase with distances from the isocenter, resulting in larger localization uncertainties for off-isocenter targets [6, 7]. Similar to the SIST, dosimetric accuracy is challenging due to the small target size [8]. The dynamic MLC-collimated delivery of doses to small off-isocenter targets makes the dosimetric accuracy even more challenging. Consequently, patient-specific quality assurance (PSQA) remains critical in ensuring the safe delivery of SIMT.

AAPM minimum practice specification MPPG 9a recommends an SRS dosimetric tolerance of 5% and a localization tolerance of 1.0 mm [9, 10]. Radiochromic film remains the gold standard for SRS verification, as it is independent of dose rate, energy and angularity, and offers sub-millimeter spatial resolution [11]. However, film measurement and processing are labor intensive and time consuming, and a 24-h delay between exposure and readout is recommended. The resources required are a barrier to routine film PSQA.

Other SIMT SRS PSQA methods have been explored [12]. Small ion chambers with an active volume ≤0.01 cc are recommended for point dose measurement, but this does not evaluate overall dose distribution [8]. An electronic portal imaging device (EPID) has the necessary high spatial resolution but is known to over-respond to low-energy photons and is dose-rate dependent [13, 14]. Further corrections are needed before an EPID can be used for SIMT SRS QA [15]. Detector arrays generally do not have the spatial resolution for SRS PSQA. For example, the Scandidos (Uppsala, Sweden) Delta4 Phantom+ has a diode spacing of 5 mm in the inner area and 10 mm in the outer region. The IBA (IBA GmbH, Germany) MatriXX Resolution has a detector spacing of 6.5 mm. The SunNuclear (Melbourne, FL) ArcCHECK QA phantom has a detector spacing of 10 mm, and the SRS MapCHECK QA phantom has a detector spacing of 2.47 mm. Without a sub-millimeter resolution, accurately measuring the dose profiles of small brain lesions is challenging [16, 17]. Recent research demonstrated the potential of using complementary metal oxide semiconductors (CMOS) as active pixel sensors for small-field dosimetry [18]. One device manufactured by IBA dosimetry (IBA GmbH), a CMOS array (myQA SRS), is now commercially available. With a pixel size of 0.4 × 0.4 mm^2^ with no inter-detector spacing, the spatial resolution approaches that of film. In addition, it provides a larger detector plane (12 × 14 cm^2^) compared to SRS MapCHECK (7.7 × 7.7 cm^2^).

Here, we report a multi-institution dosimetric commissioning of the high-resolution CMOS 2D detector array for patient-specific QA of VMAT (SIMT) stereotactic radiosurgery at Institute A and Institute B. Each institution performed identical measurements using a different CMOS 2D detector on Varian TrueBeam STx LINACs at the corresponding institutes, which provided more robust and independent validation of dosimetric comparison than at a single institute.

In addition to dose-rate, field-size, energy and beam angular dependencies, we investigated detector characteristics specific to SIMT delivery, including angular dependence in non-coplanar beams and off-axis uncertainties. Parametric investigations of off-axis distances and target sizes further explored the suitability of myQA SRS for SIMT PSQA in a wide range of scenarios. To date, only a few studies have evaluated this new CMOS detector [19–22]. Padeli *et al*. and Ashraf *et al*. evaluated its applicability to CyberKnife robotic radiosurgery QA [19, 21]. Stepanek *et al*. evaluated basic detector characteristics and their application to stereotactic body radiation therapy (SBRT) QA on an Elekta Versa HD [22]. Junis *et al*. evaluated the device to QA SBRT and SRS plans with single lesions only [20]. To our knowledge, there are no studies evaluating myQA SRS for SIMT PSQA.

## METHODS AND MATERIALS

### MyQA SRS device

**Fig. 1:**
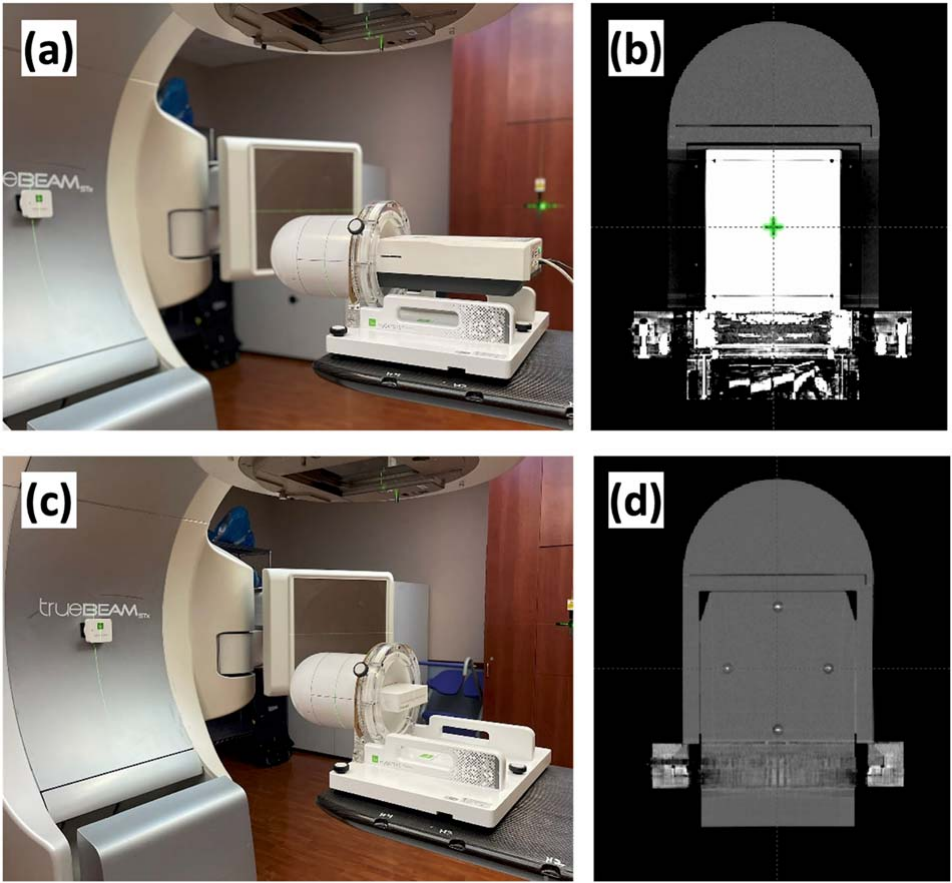
Measurement setup. The measurement setup was identical for both myQA SRS and the film dosimetry. The myQA SRS device was positioned within a cylindrical ABS phantom during dose measurement. Simultaneously, a gantry sensor, connected via Bluetooth to the acquisition software, was affixed to the gantry stem, providing a real-time gantry angle readout for angular correction (see Fig. 1a and b). The same setup was employed for film dosimetry, where four metallic pins in the film slot were used to accurately pinpoint the isocenter location with registration to the 2D detector array (Fig. 1c and d).

The myQA SRS device is a solid-state CMOS detector array with a 0.4 × 0.4 mm^2^ detector size and a spatial resolution of 0.4 mm. Volume averaging is minimal, and interpolation is unnecessary due to the absence of inter-detector spacing. The detector array measures 12 × 14 cm^2^, enabling simultaneous dose measurements from SIMT cranial SRS plan. The device is housed inside a 19.0 cm cylindrical phantom made of acrylonitrile butadiene styrene (ABS), topped by a hemispheric cap (Fig. 1a and b). The same phantom also accommodates a dedicated insert for film dosimetry (Fig. 1c and d). MyQA SRS operates at an external 24-V power source and communicates with the associated software via a 10/100/1000 Base-T Ethernet Interface.

### Absolute dose calibration

The myQA SRS device and the phantom were CT-scanned using 120 kVp, 190 mA and 1 mm slice thickness. Philips Brilliance Big Bore was used in Institute A, and Siemens SOMATOM Go.Sim was used in Institute B. The resulting DICOM data were exported to Eclipse (AAA_1610 at institution A and AAA_1506 at institution B; Varian Medical System, Palo Alto, CA) for dose calibrations. In Eclipse, the phantom body and the detector were assigned a density of 1.04 g/cc, and the active area of the detector was assigned a density of 1.0 g/cc. The absolute dose of the detector was calibrated to the absolute calibration of the linac machine, which is 1.0 cGy/MU at dmax in water at 100.0 cm SSD, using a 10.0 × 10.0 cm^2^ field size. Dose calibration files were created for 6X, 6FFF, 10FFF and 15X at their respective available dose rates. The manufacturer recommended calibrating output at 70% of the maximum dose rate for clinical use. All plans were delivered by Varian TrueBeam STx (Varian Medical System) machines at both institutions. Each institute used different myQA SRS devices, and measurements were performed on separately commissioned Varian TrueBeam STx LINACs.

### Absolute film dose calibration

The dosimetric accuracy of the myQA SRS was determined by comparison with radiochromic film, which was taken as the reference. Gamma analysis and dose difference were used as evaluation metrics. Based on its sensitivity range, we used EBT-XD films (Ashland LLC, Dublin, OH) for composite field analysis and EBT4 films (Ashland LLC) for field-by-field analysis.

The film response was calibrated by placing the film at the center of a 30 × 30 × 10 cm^3^ solid water phantom at a 5.0 cm depth and 95.0 cm SSD. Eleven jaw-defined 4 × 4 cm^2^ fields with 6X and varying dose levels, up to 120% of the maximum dose, were used to establish the film calibration curve; a 10 × 10 cm^2^ 6FFF field with a known dose was exposed as a reference to check the integrity of the calibration. Dose calculations for the center of each field were performed using the tissue-maximum ratio (TMR), collimator scatter factor (Sc) and phantom scatter factor (Sp) data. The calibration curve was taken to be valid for all the films from the same box.

The films were scanned on a 12000XL EPSON scanner with the resolution setting to 150 DPI and analyzed with myQA Patient (IBA GmbH). Multi-channel correction [23, 24] and lateral linearization correction [25] were used in the analysis process. Prior to scanning, 10–20 preview scans were performed to warm up the scanner. An 8″ × 10″ anti-Newton’s-rings glass window was used to press down and position the film onto the scan bed, reducing Newton’s rings and stabilizing the scanning light intensity [25]. A verification film with a known dose was scanned in the beginning and the end of each scanning session to validate the scanner stability. An unexposed film was scanned to correct lateral linearization [25]. The uncertainty of film dosimetry is estimated to be 2% with this film dosimetry process [11]. When a film is placed in the insert, reference points are made on the film by pin markers, allowing accurate registration with the CMOS array (Fig. 1d). Although this study did not focus on localization, registrations were limited to <1 mm/0.5°.

### Basic dosimetric characterization of the detector

#### Dose stability

Dose stability was defined as the standard deviation divided by the mean in 20-times repeated dose measurements at the central axis using 6X and 100 MU, and field size of 10 × 10 cm^2^.

#### Dose-rate dependence

Dose-rate dependence was evaluated for the 6X, 15X, 6FFF and 10FFF beams. For 6X and 15X, 100 MU was delivered at 40, 100, 200, 300, 400, 500 and 600 MU/min. For 6FFF, 100 MU was delivered at 400, 600, 800, 1000, 1200 and 1400 MU/min. For 10FFF, 100 MU was delivered at 400, 800, 1200, 1600, 2000 and 2400 MU/min. Dose-rate dependence was defined by the ratio of the CAX response to the response at a dose rate of 400 MU/min for each energy at a 0° gantry angle.

#### Field-size dependence

Field-size dependence for energies at 6X, 15X, 6FFF and 10FFF was characterized by square field irradiation defined by HDMLC and jaws, ranging from 5 to 100 mm. The MLC (jaw) combinations were 0.5 cm (0.8 cm), 1.0 cm (1.2 cm), 2.0 cm (2.2 cm), 3.0 cm (3.2 cm), 4.0 cm (4.2 cm), 6.0 cm (6.0 cm), 8.0 cm (8.0 cm) and 10.0 cm (10.0 cm). Field-size dependence was defined by the ratio of a point measurement of each field size to the 10.0 × 10.0 cm^2^ measurement. The location of the point measurement was at the point of maximum response of the smallest field (0.5 × 0.5 cm^2^). Also, 100 MU was delivered at 400 MU/min at 100 cm SDD at 0° gantry angle for all the measurements. Field-size dependence was further compared with ion-chamber measurements. CC01 detector (IBA GmbH) was used to measure output for field size from 1.0 cm (1.2 cm) to 3.0 cm (3.2 cm), and CC04 detector (IBA GmbH) was used to measure output for field size from 3.0 cm (3.2 cm) to 10.0 cm (10.0 cm) at the same setup. Field-size-dependent correction factors for CC01 were applied to ion-chamber measurements [26]. Output factors were daisy chained at field size of 3.0 cm (3.2 cm) and normalized to the measurement at field size of 10.0 cm (10.0 cm).

#### Energy dependence

Energy dependence was evaluated at 10.0 × 10.0 cm^2^ square fields for 6FFF, 6X, 10FFF and 15X beams. CAX detector’s responses were first normalized to monthly QA dosimetry so that the measurements were scaled to 100 cGy for all energies. Energy dependence was then evaluated as the response ratio of 100 cGy relative to 6FFF. Also, 100 MU was delivered at 400 MU/min at 100 cm SDD in a 10 × 10 cm^2^ field size at 0° gantry angle for each energy.

#### Angular dependence

Without angular corrections, doses were measured from gantry angles 0° to 180° in combination with couch angles at 0°, 45° and 90° for energies 6FFF, 6X, 10FFF and 15X. Also, 100 MU was delivered at 400 MU/min at 100 cm SDD in a 10 × 10 cm^2^ field size with a combination of gantry and couch angles for each energy. Angular dependence was the ratio of measured dose to TPS prediction, normalized to the data at 0° couch and 0° gantry rotations for each energy and off-axis location. A gantry angle sensor (GAS), provided by the manufacturer, was affixed to the gantry stem for a real-time readout of gantry angles.

### Patient-specific QA

A multi-institutional commissioning procedure was established as follows. First, standard plans were provided to each institution to standardize the commissioning procedures for joint commissioning. Second, each institution optionally provided clinical plans for the other institution to evaluate on their respective devices and machines.

#### Standard plans

**Fig. 2:**
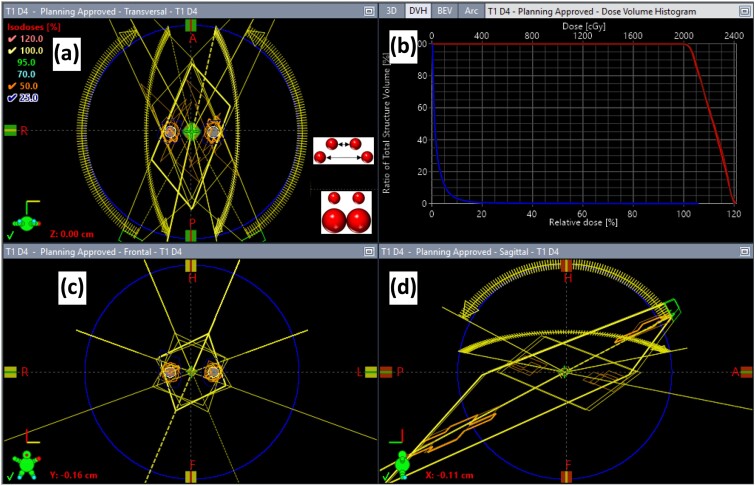
Example of a standard plan (T1D4). Standard plans have two identical spherical targets, varying in sizes and positioned at different off-isocenter distances (see inserts in Fig. 2a). Using the VMAT technique and 6FFF energy, four non-planar arcs with an isocenter equidistant to the targets delivered conformal high doses to the off-isocenter targets, achieving ~100% coverage and a sharp dose gradient (axial, coronal and sagittal dose distribution displayed in Fig. 2a, c–d). Dose–volume histogram (DVH) of the targets (top curve in red) and brain dose (bottom curve in blue) conformed to clinical criteria (Fig. 2b). Plan parameters are summarized in Fig. 2e.

Six single-isocenter two-target SRS plans were created using the Eclipse treatment planning system. Each standard plan consisted of two identical spherical lesions with diameters of 1.0 cm or 2.0 cm. The distances to the isocenter were ±1.0, ±2.0 or ±4.0 cm within a spherical phantom of diameter of 20.0 cm. Highly conformal SRS plans (20 Gy × 1) with sharp dose fall-offs were created using four non-coplanar arcs (couch angles = 67°, 20°, 293° and 240°) using the VMAT technique and 6FFF beams (Fig. 2). The average RTOG conformal index is CI_RTOG_ = 1.11 ± 0.03, and the average Paddick gradient index [27, 28] is GI = 3.18 ± 0.64. Plan files for Varian HDMLC in DICOM format are available by request. Detailed plan information is summarized in Table 1.

**Table 1:** Plan parameters

		Rx (Gy)	Target size (cc)	Off-axis locations (cm)	Couch angles (deg)	Homogeneity index
Standard plans	T1D2	20 × 1	0.51, 0.51	1.0, 1.0	67, 20, 293, 240	1.24
	T1D4	20 × 1	0.51, 0.51	2.0, 2.0	67, 20, 293, 240	1.24
	T1D8	20 × 1	0.51, 0.51	4.0, 4.0	67, 20, 293, 240	1.23
	T2D2	20 × 1	4.26, 4.26	1.0, 1.0	67, 20, 293, 240	1.26
	T2D4	20 × 1	4.26, 4.26	2.0, 2.0	67, 20, 293, 240	1.26
	T2D8	20 × 1	4.26, 4.26	4.0, 4.0	67, 20, 293, 240	1.28
Clinical plans	MSW1	16 × 1	4.46	0	0, 0	1.29
	MSW2	18 × 1	0.66, 0.41, 0.20, 0.55, 0.36	1.3, 2.0, 4.3, 4.5, 5.2	0, 0, 90, 45, 315	1.24
	MSK	21 × 1	0.62	0	0, 0, 90, 45, 315	1.25

#### Clinical plans

In addition to standard plans, three clinical plans were evaluated. One of the clinical plans consisted of five targets, delivered by two planar and three non-planar beams. The rotational plane extraction function was used to intercept the targets maximally. The other two clinical plans are single-isocenter single-target (SIST) SRS plans, one with three non-coplanar beams and the other without. The prescription dose, target size, off-isocenter locations, couch angles and homogeneity index (dmax/Rx) are summarized in Table 1. Together with SIMT SRS plans, a full range of SRS plans were delivered for PSQA evaluations. All clinical DICOM plan files were anonymized before distribution.

#### Measurement setup

The measurement setup was identical for both myQA SRS and the film dosimetry for PSQA. The myQA SRS device was positioned within a cylindrical ABS phantom during plan delivery. Simultaneously, a gantry sensor, connected via Bluetooth to the acquisition software, was affixed to the gantry stem, providing a real-time gantry angle readout for angular correction (see Fig. 1a and c). The same setup was used for film dosimetry, where four metallic pins in the film slot were used to accurately pinpoint the isocenter location with registration to the 2D detector array (see Fig. 1c and d).

### Statistical analysis

The Wilcoxon signed-rank test was used to assess the significant differences (H0: *P* > 0.05) in angular dependence between pairs of couch rotations, energies, off-axis distance, and manufacturer-provided and user-defined angular correction tables. Multivariate analysis of variance (ANOVA) was used to evaluate correlations between measured dose to dose rate, beam energies, institutions and their interactions (H0: *P* > 0.05).

## RESULTS

### Dosimetric characterization of the detector

#### Dose stability

Signal reproducibility was within ±0.5%, and output calibration was stable over 5 months.

#### Dose rate

**Fig. 3:**
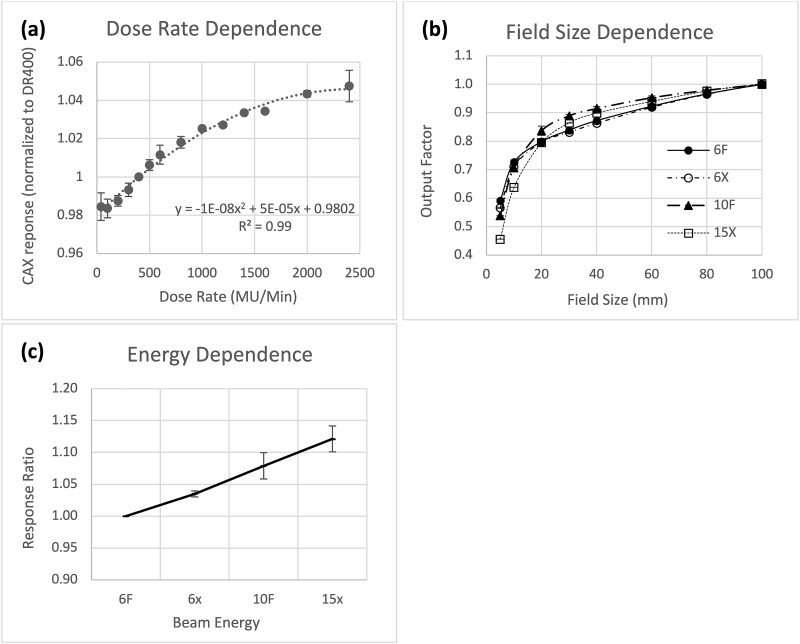
Dosimetric characteristics of the detector. (a) Dose-rate dependence: this dependence was well fitted by a second-order polynomial. *y* = −1E−08*x*^2^ + 5E−05*x* + 0.9802 (*R*^2^ = 0.99). (b) Field-size dependence: plotted for 6FFF, 6X, 10FFF and 15X beam energies. (c) Energy dependence. All figures represent the average data from both institutions.

Detector response increased approximately linearly with dose rates but began to saturate when dose rates >1000 MU/min (Fig. 3a). Dose-rate dependence was observed over a wide range using both flattened and flattened-free beams and all available energies. Dose-rate dependence was independent of energy (multivariate ANOVA, *P* > 0.5) or device (multivariate ANOVA, *P* > 0.5), so all the measurements were combined for the analysis. Normalized to the CAX detector response at a dose rate of 400 MU/min, a second-order polynomial function well describes the trend (Fig. 3a, *R*^2^ = 0.99).

#### Field size

Field-size dependence, defined by the ratio of the CAX dose ratio between the measured and the reference field (10.0 × 10.0 cm^2^), decreased approximately linearly as the field size decreased and then steeply for field size <2.0 × 2.0 cm^2^ (Fig. 3b). Both institutions displayed similar field-size dependency for field sizes between 2.0 × 2.0 cm^2^ and 10.0 × 10.0 cm^2^ for all energies (mean difference < 0.3%). The difference for field sizes of 1.0 × 1.0 cm^2^ and 0.5 × 0.5 cm^2^ increased to 1.47 ± 1.55% and 3.38 ± 1.23%, respectively. The differences to ion-chamber measurement were all within 2% for field size >1.0 × 1.0 cm^2^. Table 2 summarizes the field-size dependence between the two institutions.

**Table 2:** Field-size dependence between two institutes

FS (cm)	Institute B			Institute A			Institute variability (%)	Ion chamber			Ion-chamber comparison (%)
	6X	6FFF	10FFF	6X	6FFF	10FFF		6X	6FFF	10FFF	
10	1	1	1	1	1	1		1.00	1.00	1.00	
8	0.97	0.96	0.98	0.96	0.97	0.98	0.01	0.97	0.97	0.98	0.46
6	0.91	0.92	0.95	0.92	0.92	0.95	−0.28	0.93	0.93	0.96	1.25
4	0.86	0.87	0.92	0.87	0.87	0.91	0.09	0.88	0.89	0.93	1.50
3	0.83	0.84	0.89	0.83	0.84	0.88	0.00	0.85	0.86	0.90	1.74
2	0.79	0.8	0.84	0.8	0.8	0.83	−0.21	0.81	0.82	0.85	1.66
1	0.71	0.73	0.72	0.71	0.72	0.7	1.47	0.70	0.72	0.69	−1.65
0.5	0.58	0.6	0.55	0.55	0.59	0.53	3.38	0.47	0.49	0.43	NA

#### Energy

As beam energy increased, the response of the CMOS detector also increased. For a 100 cGy dose delivery, the signal increased by 3 ± 0%, 8 ± 2% and 12 ± 2% when delivered by 6X, 10FFF and 15X beam energies relative to 6FFF beam energy (Fig. 3c).

#### Angular dependence

The manufacturer provided an angular dependency look-up table (LUT_iba_) for correction to account for the angular dependence of the CMOS detector. Because SIMT often delivered doses to off-isocenter targets with multiple non-coplanar beams using couch rotations, we investigated whether the performance of the manufacturer-provided LUT_iba_, which corrected for detector response at the central axis at zero couch rotation, was also sufficient for detectors located at off-isocenter locations with couch rotations. Detectors located away from the isocenter should observe incident angles different from detectors located on the isocenter at the same gantry rotation.

#####  

**Fig. 4:**
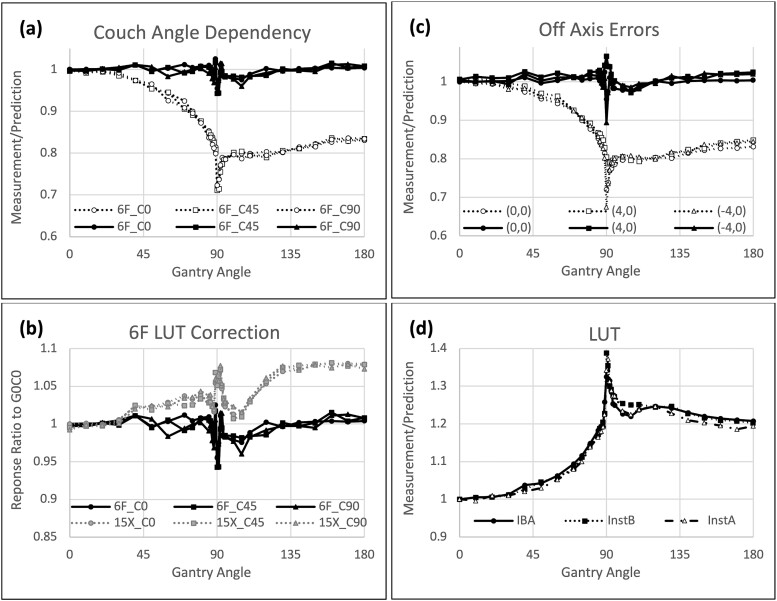
Angular dependence of the detector. (a) Measured dose to predicted dose at isocenter: plotted as a function of gantry angles from 0° to 180° at three couch rotations of 0°, 45° and 90°, before (open markers) and after (filled markers) angular corrections. An example of 6FFF beam energy. (b) Residual dose errors following angular correction: shown as a function of gantry angles from 0° to 180° at three couch rotations of 0°, 45° and 90° for 6FFF (black) and 15X beam (gray) energies. (c) Measured dose to predictor dose at isocenter and off-isocenter locations: presented as a function of gantry angles from 0° to 180°, averaged over three couch rotations of 0°, 45° and 90° before (open markers) and after (filled markers) angular corrections. An example of 6FFF beam energy. (d) Comparison of manufacturer-provided angular correction look-up table (LUT) and user-defined empirical angular corrections (Institute A and Institute B). An example of 6FFF energy.

*(a) On couch rotation.* Three couch angles at 0°, 45° and 90° were used to examine angular dependency on couch rotations during gantry rotations. Referenced to gantry 0° and couch 0°, the ratio of measured to prediction doses at the central axis decreased as the gantry moved away from 0°, with a maximum error at gantry 90°. Statistical analyses showed no differences in the characteristic curves of angular dependency at different couch angles (Table 3, columns 2–4, Wilcoxon signed-rank test, *P* > 0.05), suggesting that angular dependence is radially symmetric to couch rotations (Fig. 4a dashed lines).

**Table 3:** Angular dependence tests

	Wilcoxon signed-rank test											
	(C0, C45)	(C0, C90)	(C45, C90)	(6F, 6X)	(6F, 10F)	(6F, 15x)	(10F, 15x)	(0, +4)	(0, −4)	(+4, −4)	(IBA, InstA)	(IBA, InstB)
h	0	0	0	0	1	1	1	1	1	1	0	0
*P*	0.05	0.05	0.67	0.36	3.5E−06	2.1E−06	3.9E−06	3.4E−06	7.3E−03	1.1E−03	0.08	0.79

The Wilcoxon signed-rank test was conducted to assess the significance (*P* < 0.05) of differences in angular dependence among various factors, including pairs of couch rotations (columns 2–4), energies (columns 5–8), off-axis distance (columns 9–11), and manufacturer-provided and user-defined LUTs (columns 12–13).

#####  

*(b) On beam energy.* Before angular correction, the average errors in detector response to a full range of gantry angle rotations at CAX and couch 0° were −12.1 ± 8.1%, −12.1 ± 8.0%, −9.6 ± 7.2% and −8.7 ± 6.4% of the TPS dose predictions, with maximum errors of −28.6%, −27.3%, −24.5% and −20.7% near gantry 90° for beam energies of 6FFF, 6X, 10FFF and 15X, respectively (Fig. 4a dashed line).

Applying the manufacturer-provided angular corrections, the average errors were reduced to −0.21 ± 1.5%, −0.16 ± 1.5%, 2.9 ± 1.8% and 3.9 ± 2.6% of the predictions, with maximum error of −5.7%, −3.9%, +4.5% and +7.7% near gantry 90° for beam energies of 6FFF, 6X, 10FFF and 15X, respectively (Fig. 4a solid lines).

Although detector responses following angular correction had average errors <4% for all energies, the Wilcoxon signed-rank test showed that LUT should be energy dependent (Table 3, columns 5–8, Wilcoxon signed-rank test, *P* < 0.05). Manufacturer-provided LUT was suitable for correcting angular dependency for 6X and 6FFF, but different LUTs may be needed for 10FFF and 15X separately (Table 3, columns 5–8, Fig. 4b) to minimize errors.

#####  

*(c) On off-isocenter distance.* Angular dependency of detectors at off-axis positions (+4.0 cm, 0 cm) and (−4.0 cm, 0 cm) were compared to those located at the isocenter (0 cm, 0 cm). Although the ratio between measured and predicted doses did not display a salient change (Fig. 4c), statistical analysis showed a small but significant difference in the angular dependency curves (Table 3, columns 9–11, Wilcoxon signed-rank test, *P* < 0.05). Applying the same LUT correction, dose errors averaged over the three couch rotations at 0°, 45° and 90° and a half-arc gantry rotation were −0.02%, 1.1% and 0.6% for detectors at (0 cm, 0 cm), (+4.0 cm, 0 cm) and (−4.0 cm, 0 cm), demonstrating a detector’s sensitivity to small changes of incident angles caused by off-isocenter locations.

#####  

*(d) Compared to user-defined LUT.* A user could empirically commission a site-specific LUT by correcting the measured dose to the predicted dose with a correction factor at each gantry angle (Fig. 4d). Our data showed no statistical difference between the manufacturer-provided LUT and user-defined LUTs (Table 3, columns 12–13, Wilcoxon signed-rank test, *P* > 0.05).

### Dosimetric accuracy comparison to film dosimetry

**Fig. 5:**
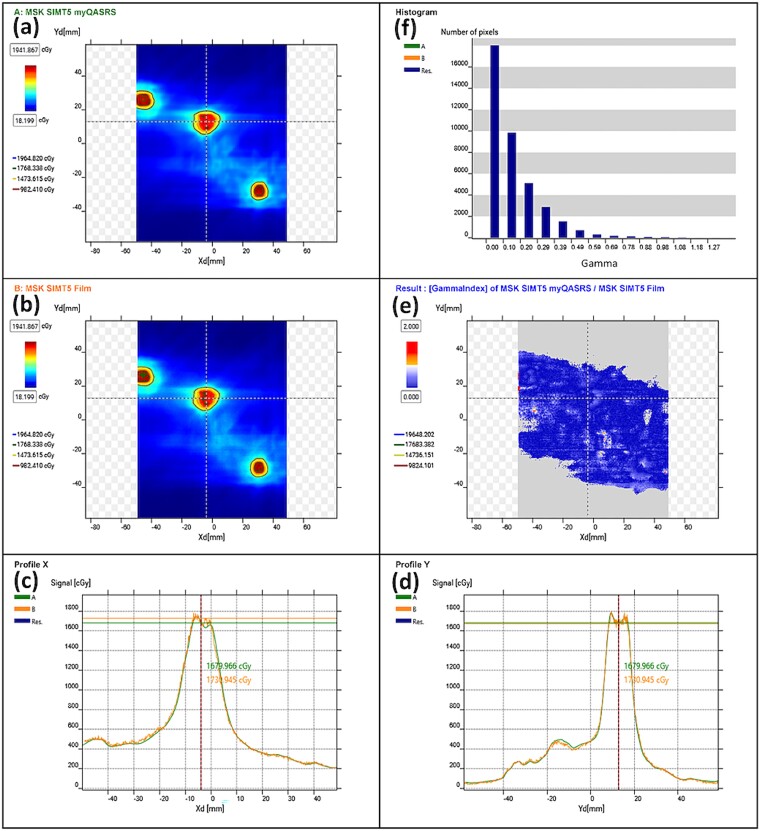
Dose correlation between myQA SRS and film. This example features a single-isocenter and 5-target clinical case (MSW2) measured at Institute A. (a) Dose distribution measured by myQA SRS. (b) Dose distribution measured by film. (c) Dose profile along the *x*-axis comparing myQA SRS (green) and the film (orange). (d) Dose profile along the *y*-axis comparing myQA SRS (green) and the film (orange). (e) Gamma index distribution at local 1%/1 mm criteria with a 10% threshold. (f) Histogram of the gamma index. At 50% dose threshold, the average dose differences between myQA SRS and film are 0.1% (left), 0.6% (middle) and 0.7% (right), respectively. Distance-to-agreement (DTA) pass rate with criteria at 1.0 mm and 10% threshold is 99.7%.

Gamma index analyses were performed in the IBA myQA software v. 2.17.11.0 (see Fig. 5 for an example) using the recommended metrics for regular QA and commissioning [29]. At Institute A, the average gamma pass rates between myQA SRS and film in all nine plans (six standard and three clinical plans) were 99.9 ± 0.03% and 99.8 ± 0.36% for the criteria of 3%/2 mm and 1%/1 mm, respectively, with a 10% threshold. At Institute B, the average gamma pass rates were 99.9 ± 0.03% and 99.8 ± 0.19% for the same criteria. At clinical PSQA gamma pass criteria of 3%/2 mm at a 10% threshold, all plans passed >99.9% at both institutions.

In addition to composite plan gamma index analyses, field-by-field gamma index analyses (examples for T1D8_F1, F2, F3, F4) also showed high dosimetric correlation with the film. At Institute A, the average gamma index pass rates between myQA and film all four fields from T1D8 plan were 99.9 ± 0.05% and 99.0 ± 0.75% for the criteria of 3%/2 mm and 1%/1 mm, respectively, with a 10% global threshold. At Institute B, the average gamma index pass rates between myQA and film all four fields from T1D8 plan were 99.8 ± 0.21% and 98.8 ± 0.26% for the criteria of 3%/2 mm and 1%/1 mm, respectively, with a 10% global threshold. Even by a much more stringent criteria of local 1%/1 mm at a 10% threshold, the average pass rate was 97.7 ± 1.19% (summarized in Table 4). Furthermore, dose differences were evaluated between myQA SRS and film dosimetry. On average, the dose ratio of myQA SRS to film is 0.2 ± 1.0% at Institute A and 0.5 ± 1.2% at Institute B.

**Table 4:** Gamma pass rate between MyQA SRS and film

		Institute A				Institute B			
		3%/2 mm G	3%/2 mm L	1%/1 mm G	1%/1 mm L	3%/2 mm G	3%/2 mm L	1%/1 mm G	1%/1 mm L
Standard plans	T1D2	100	100	100	99.8	100	100	100	99.4
	T1D4	100	100	99.8	99.8	100	100	100	99.9
	T1D8	100	99.7	99.7	98.9	100	100	99.6	99.4
	T2D2	100	100	99.9	99.2	100	100	100	99.4
	T2D4	100	100	99.5	98.7	99.9	99.5	99.7	97.3
	T2D8	99.9	99.7	98.9	95.5	100	100	100	99.8
Clinical plans	MSW1	100	100	100	99.5	100	100	100	100
	MSW2	100	100	100	99.8	100	100	99.6	99.1
	MSK	100	100	100	100	100	100	99.7	99.7
AVERAGE		100 ± 0.0	99.9 ± 0.1	99.8 ± 0.4	99.0 ± 1.4	100 ± 0.0	99.9 ± 0.2	99.8 ± 0.2	99.3 ± 0.8
Field by field	T1D8_F1	100	99.9	99.8	99.4	100.0	100.0	99.5	97.2
	T1D8_F2	100	100	98	97.9	100.0	99.7	99.2	97.8
	T1D8_F3	100	100	99.2	99.1	99.7	99.5	98.6	98.0
	T1D8_F4	99.9	99.9	98.9	98.5	99.6	99.4	97.8	95.6
AVERAGE		100 ± 0.0	100 ± 0.1	99.0 ± 0.7	98.7 ± 0.7	99.8 ± 0.2	99.7 ± 0.3	98.8 ± 0.8	97.1 ± 1.1

Gamma pass rates were computed by comparing myQA SRS to film of the composite doses of the same plans either measured at Institute A or Institute B, using criteria of 3%/2 mm and 1%/1 mm at 10% thresholds with local and global. Additional field-by-field measurements were conducted specifically for plan T1D8.

There was no significant difference in gamma pass rates between composite and field-by-field dose analysis (Wilcoxon signed-rank test, *P* = 0.26), and no significant difference between Institute B and Institute A (Wilcoxon signed-rank test, *P* = 0.52).

## DISCUSSION

A newly available CMOS 2D detector was tested for SIMT PSQA. Compared to other SRS QA devices, such as SRS MapCHECK or MapCHECK 3, this new device provides both a sub-millimeter resolution of 0.4 mm and a sufficiently large detector plane of 12 × 14 cm^2^ for SIMT PSQA. It enabled users to simultaneously record a planar dose distribution without having to shift the device to chase far-away targets. Dosimetric characterization showed that the detector was energy and dose-rate dependent, which is consistent with other reports of the same device [20–22]. The manufacturer has recommended calibrating the dose at 70% of the maximum dose rate for each energy. However, the dose rates of a plan can be previewed at the MLC’s control points. All the standard and clinical plans tested in this study were delivered using 6FFF at 1400 MU/min dose rate, and hence, we used the dose calibration for 6FFF at 1400 MU/min for the dose report compared with film. Using the dose calibrations at the plan dose rates may improve dose accuracy by up to 2.0%, consistent with prior studies [20].

Field-size dependence was affected by small-field dosimetric challenges, where a rapid dose reduction results from the lack of lateral charged particle equilibrium. Hence, the characteristic curves of the output factors were both field-size and energy dependent (Fig. 2b). Our results were consistent with previous studies of the same device, where the authors further verified the output factors using ion-chamber measurements [20, 22]. Between Institute A and Institute B, the output started to diverge from 0% at a field size of 2.0 × 2.0 cm^2^ to up to 3.4% at 0.5 × 0.5 cm^2^ field size (Table 2). The difference in the output factors illustrated the detectors’ sensitivity to machine differences, most likely in the MLC dosimetric leaf gap, focal spot size and shape, and setup uncertainties.

To our knowledge, this is the first report of testing the applicability of myQA SRS for SIMT PSQA. SIMT delivery is unique from other SRS or SBRT delivery because high-dose regions are located away from the isocenter. In addition, multiple couch rotations must be employed to create a highly conformal plan and to reduce high brain dose volumes. Prior study showed that the shape of gantry angular dependence is couch-angle dependent [20]. However, we found the shape of gantry angular dependence to be independent of couch angles. In our study, we first ratio the detector response to predicted dose, to normalize differential detector depths at different couch angles. This step allowed us to separate the effect of gantry angle dependence from phantom geometry. As a result, we found gantry angle dependence to be dependent on energy and off-isocenter distances, but not on couch rotations.

Noticeably, even following angular correction, residual dosimetric errors remained highest at gantry angles between 85° and 95°, where correction factors peaked. The sharp gradient of LUT near gantry 90° (Fig. 3d) renders the corrections near the peak particularly susceptible to setup, machine calibration and gantry sensor readout uncertainties. However, as SIMT SRS used the VMAT technique with multiple gantry angles, the residual dosimetric errors at the central axis average to near zero for a full range of gantry rotation (e.g. <0.2% for 6FFF).

Geometrically, a detector located at a 4.0 cm distance from the isocenter would observe an incident angle of 2.3° instead of 0° from a 100.0 cm SDD, as a photon beam coming from a 0° gantry angle. These small but statistically significant differences in angular dependency found in off-isocenter detectors could result in ~1% dose errors. It also created a dose-error asymmetry when a half-arc gantry rotation is used instead of a full arc. Consequently, SIMT PSQA is affected by a systemic off-axis dosimetric uncertainty. Users should use clinical judgment when assessing targets far away from the isocenter.

Excellent correlations were found between myQA SRS and films, similar to single-target SBRT plans [22]. The gamma index pass rates were 99.9 ± 0.03% at 3%/2 mm using 10% threshold, and 99.2 ± 1.1% at local 1 mm/1% using 10% threshold averaged over all plans and across institutes. In addition, dose differences between film and myQA SRS were averaged at 0.4 ± 1.3%, well within the film measurement uncertainty of 2%. Based on our results, the manufacturer’s recommended HU overwrites provided an excellent dosimetric agreement between myQA SRS and films.

Furthermore, the focus here is on the dosimetric accuracy of the device. The dosimetric equivalence between the CMOS 2D detector array and the film measurement (the PSQA gold standard) has been established. The results demonstrated that the array can be used for PSQA and commissioning, which will significantly reduce the workload of clinical physicists and improve patient safety. Although the dosimetric comparison between the two institutes’ CMOS 2D detector and the treatment planning systems warrants further investigation, it is beyond the current study’s scope. Further investigation will also be performed to investigate the localization accuracy and assess the efficacy of the device to be used as a tool for the end-to-end test.

In conclusion, with appropriate dose calibrations and angular corrections, a CMOS 2D detector, myQA SRS, has demonstrated its potential as a reliable tool for PSQA in SIMT SRS. It offers high spatial resolution, sufficiently large detector plans and ease of use in a clinical setting, especially for SIMT QA. The excellent dosimetric agreement between myQA SRS and film was consistent between the two institutions, further validating the dosimetric accuracy and reproducibility of the myQA SRS system. It offers a timely alternative to film dosimetry for SRS commissioning and SIMT PSQA.

## Abbreviations

2D, two-dimensional; AAA, anisotropic analytical algorithm; AAPM, American Association of Physicists in Medicine; ABS, acrylonitrile butadiene styrene; CAX, central axis; CI, conformal index; CMOS, complementary metal oxide semiconductor; DICOM, Digital Imaging and Communications in Medicine; dmax, dose maximum; DR, dose rate; DPI, dots per inch; DVH, dose volume histogram; EPID, electronic portal imaging device; FFF, flattening filter free; GAS, gantry angle sensor; GI, gradient index; HDMLC, high-definition multi-leaf collimator; LINAC, linear accelerator; LUT, look-up table; MLC, multi-leaf collimator; MPPG, Medical Physics Practice Guideline; Institute A, Memorial Sloan-Kettering Cancer Center; Institute B, Mount Sinai West; MU, monitor unit; PSQA, patient-specific quality assurance; RTOG, Radiation Therapy Oncology Group; QA, quality assurance; SIMT, single isocenter multiple target; SIST, single isocenter single target; SRS, stereotactic radiosurgery; TB, TrueBeam; TMR, tissue maximum ratio; Sc, scatter factor; Sp, phantom scatter factor; SSD, source surface distance; SDD, source detector distance; VMAT, volumetric modulated arc therapy
